# Artificial intelligence-enhanced 12-lead electrocardiography for identifying atrial fibrillation during sinus rhythm (AIAFib) trial: protocol for a multicenter retrospective study

**DOI:** 10.3389/fcvm.2023.1258167

**Published:** 2023-10-11

**Authors:** Yong-Soo Baek, Soonil Kwon, Seng Chan You, Kwang-No Lee, Hee Tae Yu, So-Ryung Lee, Seung-Young Roh, Dong-Hyeok Kim, Seung Yong Shin, Dae In Lee, Junbeom Park, Yae Min Park, Young Ju Suh, Eue-Keun Choi, Sang-Chul Lee, Boyoung Joung, Wonik Choi, Dae-Hyeok Kim

**Affiliations:** ^1^ Division of Cardiology, Department of Internal Medicine, Inha University College of Medicine and Inha University Hospital, Incheon, Republic of Korea; ^2^ DeepCardio Inc., Incheon, Republic of Korea; ^3^ School of Computer Science, University of Birmingham, Birmingham, United Kingdom; ^4^ Division of Cardiology, Department of Internal Medicine, Seoul National University College of Medicine and Seoul National University Hospital, Seoul, Republic of Korea; ^5^ Department of Preventive Medicine, Yonsei University College of Medicine, Seoul, Republic of Korea; ^6^ Department of Cardiology, Ajou University School of Medicine, Suwon, Republic of Korea.; ^7^ Division of Cardiology, Department of Internal Medicine, Yonsei University College of Medicine, Seoul, Republic of Korea; ^8^ Division of Cardiology, Korea University Guro Hospital, Seoul, Republic of Korea; ^9^ Division of Cardiology, Ewha Womans University Seoul Hospital, Seoul, Republic of Korea; ^10^ Cardiovascular and Arrhythmia Centre, Chung-Ang University Hospital, Chung-Ang University, Seoul, Republic of Korea; ^11^ Division of Cardiology, Korea University Ansan Hospital, Ansan, Republic of Korea; ^12^ Division of Cardiology, Chungbuk National University Hospital, Cheongju, Republic of Korea; ^13^ Division of Cardiology, Ewha Womans University Mokdong Hospital, Seoul, Republic of Korea; ^14^ Division of Cardiology, Department of Internal Medicine, Gachon University Gil Medical Center, Incheon, Republic of Korea; ^15^ Department of Biomedical Sciences, Inha University College of Medicine and Inha University Hospital, Incheon, Republic of Korea; ^16^ Department of Computer Engineering, Inha University, Incheon, Republic of Korea; ^17^ Department of Information and Communication Engineering, Inha University, Incheon, Republic of Korea

**Keywords:** atrial fibrillation, electrocardiography, artificial intelligence, deep learning, neural networks

## Abstract

**Introduction:**

Atrial fibrillation (AF) is the most common arrhythmia, contributing significantly to morbidity and mortality. In a previous study, we developed a deep neural network for predicting paroxysmal atrial fibrillation (PAF) during sinus rhythm (SR) using digital data from standard 12-lead electrocardiography (ECG). The primary aim of this study is to validate an existing artificial intelligence (AI)-enhanced ECG algorithm for predicting PAF in a multicenter tertiary hospital. The secondary objective is to investigate whether the AI-enhanced ECG is associated with AF-related clinical outcomes.

**Methods and analysis:**

We will conduct a retrospective cohort study of more than 50,000 12-lead ECGs from November 1, 2012, to December 31, 2021, at 10 Korean University Hospitals. Data will be collected from patient records, including baseline demographics, comorbidities, laboratory findings, echocardiographic findings, hospitalizations, and related procedural outcomes, such as AF ablation and mortality. De-identification of ECG data through data encryption and anonymization will be conducted and the data will be analyzed using the AI algorithm previously developed for AF prediction. An area under the receiver operating characteristic curve will be created to test and validate the datasets and assess the AI-enabled ECGs acquired during the sinus rhythm to determine whether AF is present. Kaplan–Meier survival functions will be used to estimate the time to hospitalization, AF-related procedure outcomes, and mortality, with log-rank tests to compare patients with low and high risk of AF by AI. Multivariate Cox proportional hazards regression will estimate the effect of AI-enhanced ECG multimorbidity on clinical outcomes after stratifying patients by AF probability by AI.

**Discussion:**

This study will advance PAF prediction based on AI-enhanced ECGs. This approach is a novel method for risk stratification and emphasizes shared decision-making for early detection and management of patients with newly diagnosed AF. The results may revolutionize PAF management and unveil the wider potential of AI in predicting and managing cardiovascular diseases.

**Ethics and dissemination:**

The study findings will be published in peer-reviewed publications and disseminated at national and international conferences and through social media. This study was approved by the institutional review boards of all participating university hospitals. Data extraction, storage, and management were approved by the data review committees of all institutions.

**Clinical Trial Registration:**

[cris.nih.go.kr], identifier (KCT0007881).

## 1. Introduction

Atrial fibrillation (AF), the most common arrhythmia, is a significant public health problem with an increasing worldwide prevalence, leading to higher healthcare costs and increased mortality, as well as the risk of ischemic stroke, heart failure, and dementia in patients (1–5).

However, it is difficult to identify AF, particularly paroxysmal AF (PAF), from electrocardiography (ECG) acquired during sinus rhythm (SR). Various screening methods, from palpation or auscultation to handheld ECG, patch-type, and implantable loop recorder, increase AF detection (5). Despite these efforts, AF remains underdiagnosed and undertreated (6). Thus, it is important to emphasize screening and early detection, with or without symptoms, to prevent cardiovascular outcomes associated with AF. The EAST AF NET trial demonstrated that early rhythm control reduced cardiovascular outcomes in patients with early AF (7, 8).

While ECG interpretation typically requires specialized knowledge and experience, AI-enhanced ECGs, using deep neural networks, have the capability to detect complex signals and patterns that elude human experts. The AI-enhanced ECG is a powerful non-invasive biomarker that has considerable clinical potential within this emerging specialized field (9, 10). Recently, machine learning and artificial intelligence (AI) have shown promising results in identifying individuals with previous AF episodes from those with SR (11, 12). A deep learning-based algorithm using a 12-lead ECG has been developed, demonstrating high accuracy in detecting AF during SR. However, external validation is needed, and multicenter studies are essential to establish its clinical utility and association with clinical outcomes.

This multicenter study aims to validate and externally verify an AI-enhanced ECG for predicting PAF during sinus rhythm. This study also aims to investigate whether this algorithm is associated with AF-related clinical outcomes including hospitalization, related procedure outcomes, and mortality.

### 1.1. Hypothesis

We hypothesized that an AI-enhanced electrocardiogram (ECG) algorithm for predicting PAF during sinus rhythm would demonstrate a significant association with AF-related clinical outcomes, including hospitalization rates, procedural success, and mortality, when validated and externally verified across multiple tertiary hospitals. This hypothesis implies that the application of this advanced AI-enhanced ECG algorithm may lead to improved PAF prediction, enhanced patient care, and optimization of clinical management strategies.

## 2. Methods and analysis

### 2.1. Study design and the AI modeling process

**Figure 1:**
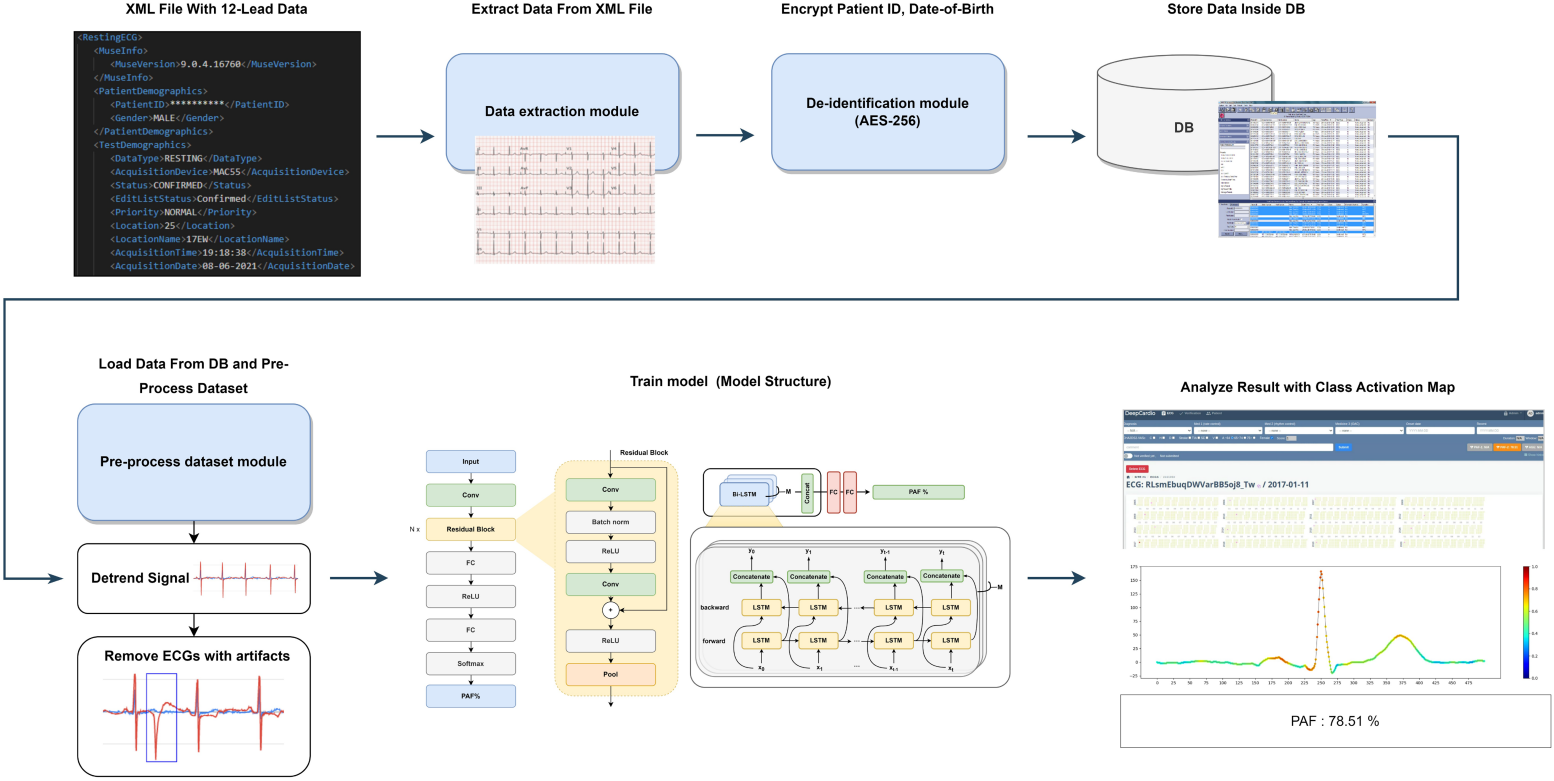
Study flowchart for the artificial intelligence modeling process.

This study is a retrospective cohort analysis based on over 50,000 12-lead ECGs. It includes ECGs displaying SR in both patients with PAF and healthy individuals, with each group comprising over 25,000 cases. Data from Inha University Hospital were collected between May 2020 and December 31, 2021, to ensure that the data are independent and do not overlap with those used in our previous research, thereby avoiding potential overfitting and bias (12). Additionally, data from nine other institution in South Korea were collected between November 1, 2012, and December 31, 2021. The study will be conducted in a multicenter setting and patient data, including baseline demographics, comorbidities, laboratory findings, echocardiographic parameters, hospitalization, and AF-related clinical outcomes, will be collected from the medical records. The de-identified ECG data will be analyzed using our AF prediction AI algorithm (SmartECG-AF®), which uses data encryption and anonymization. As we described in our previous paper on the model we developed, we will extract and analyze XML data from the MUSE data management system to minimize artifacts (GE Healthcare, Chicago, IL, USA). The original ECG recordings are measured on 12 leads. However, due to the data storage methodology of the device, only data from eight leads are stored excluding Lead III, aVR, aVL, and aVF. Nevertheless, data from these four leads can be calculated with simple arithmetic expressions, and it is a common practice to approximate the data with these operations. Therefore, this study will utilize only the eight measured signals of leads I, II, V1, V2, V3, V4, V5, and V6. The signals will be measured for 10 s on each lead simultaneously. As a result, eight one-dimensional arrays for each XML file will be obtained. As illustrated in Figure 1, the extracted data will be securely stored within the database, employing Advanced Encryption Standard (AES)-256 encryption on personal information. Subsequently, signal data preprocessing will eliminate cases with potential noise artifacts. A procedure will be implemented to exclude abnormal changes in the signal data that could be interpreted as noise artifacts. The AI algorithm will use Bidirectional Long Short-Term Memory (Bi-LSTM) and a Convolutional Neural Network (CNN), each with unique characteristics. Bi-LSTM models excel in handling sequential data, making them particularly suitable for tasks involving time series or axis such as ECG. The algorithm uses a softmax function in its final layer to produce a probability distribution for multiple classes. This “AF probability” is then presented as a confidence measure for predicting the likelihood of AF during SR. Furthermore, CNNs will be prominently employed for processing grid-like data such as images. To facilitate interpretation, a Class Activation Map (CAM) will be adopted as an explainable AI to interpret the class prediction result. CAM will allow the identification of the most influential regions within the input data that contribute to the class prediction. Finally, the output of the model will yield the probability of AF (Figure 1).

### 2.2. Inclusion criteria for center and patient

Our study included university institutions with ECG raw data in the XML format, staffed by cardiologists specialized in electrophysiology, aiming to minimize artifacts and optimize artificial intelligence performance through enhanced data quality and labeling. Study participants include patients aged 18 years and above diagnosed with PAF with a minimum follow-up period of one year. The initial ECG recording with AF is denoted as the index ECG, and subsequent ECGs with SR will be obtained. According to the guidelines, PAF is defined as AF episodes lasting less than 48 h, resolving spontaneously within 7 days, or terminating within 48 h following electrical or pharmacological cardioversion (5). Patients diagnosed with PAF will be initially identified through Electronic Medical Records (EMRs) and Clinical Data Warehouse (CDW) systems in their hospital. Cardiologists who are specialists in electrophysiology at each participating institution will review the patients according to the above definition. An SR ECG will be obtained 1 month prior to the index AF date and repeated after the index AF date (11, 12). To reduce the potential for distorted results in external validation, it is necessary to limit the number of ECGs per patient to five or fewer. Consistent with our previous research exploring the impact of electrical and structural remodeling on the “window period”, we will select ECGs that are temporally close to either the most recent or remote AF onset time. The control group will consist of individuals with normal sinus rhythm (NSR) ECGs who have no diagnosis of PAF or any recorded AF rhythm (healthy-NSR) after reviewing EMRs, CDW, and ECGs. The healthy-NSR group will be mainly selected from those who have undergone annual health check-ups or screenings at the respective hospitals for a minimum of two years to ensure their accurate classification.

### 2.3. Exclusion criteria

**Figure 2:**
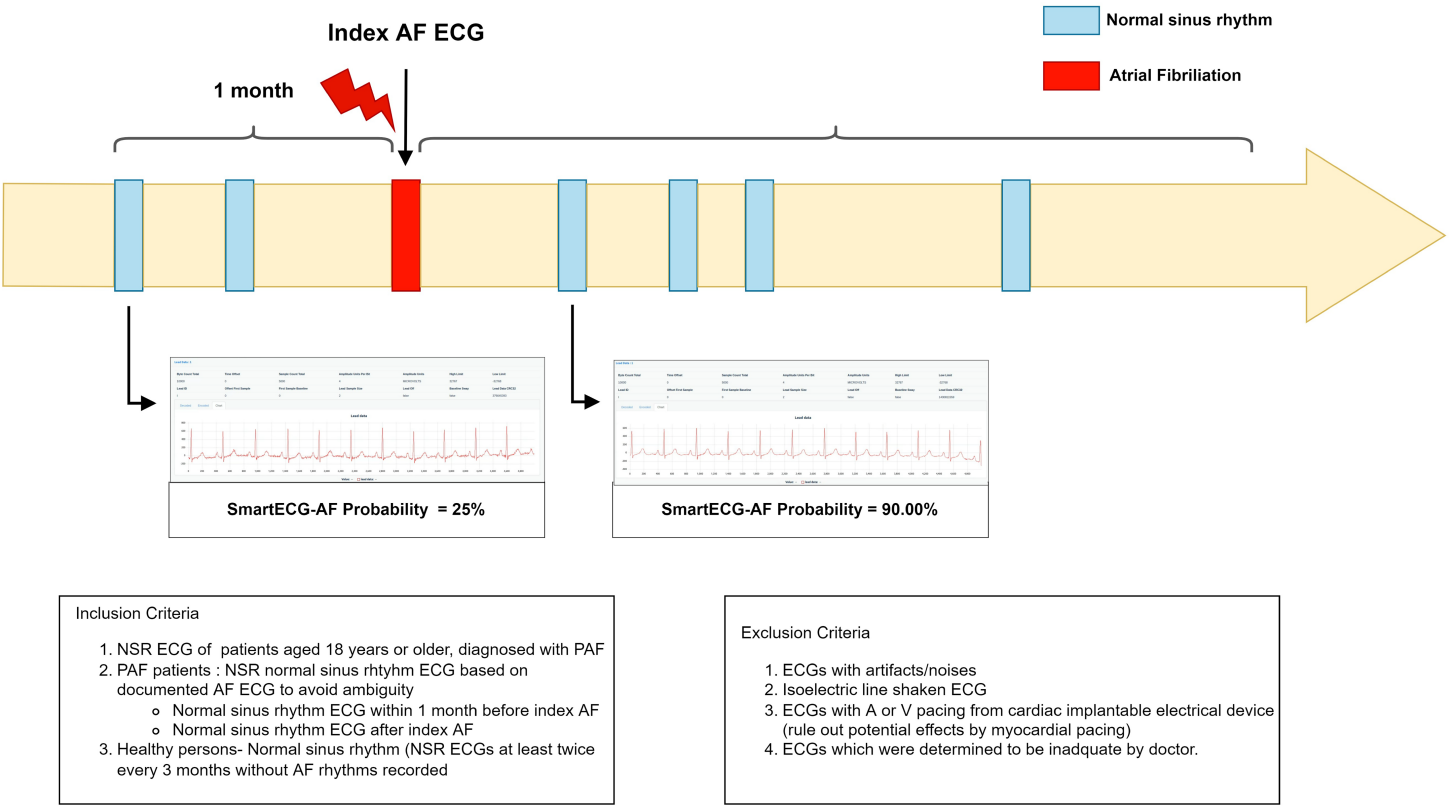
Summary diagram of inclusion and exclusion.

Patients with incomplete or missing baseline demographic data, comorbidities, laboratory findings, echocardiographic findings, and hospitalization-related procedure outcomes are excluded from the study. Patients with pacemakers, implantable cardioverter defibrillators, or other implanted devices that may interfere with ECG data acquisition are excluded to eliminate the potential effects of myocardial pacing. Patients with a history of permanent AF are also excluded from the study in accordance with the protocol. Other exclusion criteria include ECGs with artifacts or noise, isoelectric line-shaken ECGs, and ECGs deemed inadequate by physicians. Figure 2 summarizes the inclusion and exclusion criteria.

### 2.4. Study variables

We will examine the patients' basic demographic characteristics, family history, and CHA2DS2-VASc and HAS-BLED scores. The index date for AF diagnosis is defined as the date of the first documented AF ECG, and the most recent AF event since registration is defined as the latest AF date. The window period is defined as the difference between the registration date and the recent AF date. If there are blood test results within 1 month and echocardiography findings within 3 months of the registration date of the ECG, they will be additionally obtained. Information about continuous medication for more than 3 months will also be recorded. The records of hospitalizations or emergency departments will be investigated, as well as deaths after the acquisition date. Medical history of direct current cardioversion (DCCV) or AF ablation procedures will also be examined to investigate the relationship between the AI-enhanced ECG-based AF score (SmartECG-AF®) and response to AF treatment. Information collected from the patients enrolled in this trial is presented in Table 1.

**Table 1:** Information collected from enrolled patients.

Variable	Baseline	3 months	6 months	9 months	12 months (Follow-up)
Baseline demographics (age, sex, height, weight, social habit (alcohol, smoking)	X				
Family history	X				
Comorbidities (CHADS-VASc & HAS-BLED risk factors)	X				
AF index date*	X				
AF recent date^+^	X				
Window period^#^	X				
12-lead ECG	X				
TTE findings (EF, LA size, E/e’, valve disease etc.)	X				
Laboratory findings (CBC, electrolyte, creatinine, BUN, NT-proBNP, fasting plasma triglycerides, HDL cholesterol, total cholesterol, CRP)	X				
Medications (NOAC, warfarin, anti-hypertensive drugs, beta blocker, AAD)	X				
AF procedure (AFCA, Cryoablation, DCCV): SR maintenance at 6 months and 12 months	X	X	X	X	X
Hospitalization or visit for emergency department after acquired ECG (heart failure, transient ischemic attack or stroke, systemic thromboembolic event, intracranial hemorrhage or gastrointestinal bleeding and coronary artery disease or percutaneous coronary intervention)	X				X

AAD, antiarrhythmic drug; AF, atrial fibrillation; AFCA, atrial fibrillation catheter ablation; BUN, blood urea nitrogen; CHADS-VASc, Congestive heart failure, Hypertension, Age, Diabetes mellitus, previous Stroke/transient ischemic attack; CRP, C-reactive protein; DCCV, direct current cardioversion; ECG, electrocardiogram; EF, ejection fraction; HAS-BLED, Hypertension, Abnormal renal and liver function, Stroke, Bleeding, Labile international normalized ratio, Elderly, and Drugs or alcohol; HDL, high-density lipoprotein; LA, left atrium; NOAC, non-vitamin K oral anticoagulant; NT-proBNP, N-terminal pro–B-type natriuretic peptide; SR, sinus rhythm, TTE, transthoracic echocardiogram.

^*^ AF index date represents the day when AF was first diagnosed.

^+^ AF recent date denotes the day of the most recent occurrence of AF, measured from the acquisition date of the SR ECG.

^#^ The window period is quantified as the interval between the registration date and the recent AF date.

### 2.5. Follow-up

All patients with a follow-up observation period of at least one year will be included in the study. A sub-analysis will be performed to record DCCV or AF ablation procedures to investigate the relationship between the AF score by AI-enhanced ECG and AF procedure. Subsequently, SR maintenance or AF recurrence at 3, 6, 9, and 12 months will be determined.

### 2.6. Objectives

The primary objective of this multicenter retrospective cohort study is to externally validate a deep-learning-based algorithm (SmartECG-AF®) for predicting PAF during SR using 12-lead ECG data. The secondary objectives are to evaluate the impact of AI-enhanced ECG on AF-related clinical outcomes and to assess its relationship with key clinical endpoints, including all-cause mortality, cardiovascular-related mortality, cardiovascular-related hospitalization, and major adverse cardiovascular events. A sub-analysis will be conducted to investigate the relationship between AF scoring and maintenance of SR status at 1, 3, 6, and 12 months in those who received rhythm control treatment by AF catheter ablation, cryoablation, or DCCV. The risk score by AI-enhanced ECG will be used to examine the relationship between AF progression, AF duration, CHA2DS2-VASc score, and HAS-BLED score. Medications (such as non-vitamin K oral anticoagulant (NOAC) and antiarrhythmic drug (AAD)) and laboratory findings (complete blood count, electrolytes, creatinine, blood urea nitrogen, N-terminal pro b-type natriuretic peptide, fasting plasma triglycerides, high density lipoprotein cholesterol, total cholesterol, and C-reactive protein and transthoracic echocardiogram findings (ejection fraction, left atrial size, E/e') will be analyzed according to the AF risk determined by AI analysis.

### 2.7. Statistical analysis

The necessary sample size for this study was determined using power analysis. Based on an AUC value of 0.78 in our previous study, we aim for an AUC of 0.80 in the current study (12). Our study sample of 50,000 enrollees including 25,000 in the PAF-NSR group (positive group) and 25,000 in the healthy-NSR group (negative group) can achieve a statistical power of over 99% to detect a difference between AUC_0_ = 0.78 under the null hypothesis and AUC_1_ = 0.80 under the alternative hypothesis using a two-sided z-test at a significance level of 0.05.

Categorical variables are presented as frequencies and percentages, while continuous variables are summarized as means and standard deviations or medians and interquartile ranges. To determine the distribution of the continuous variables and the appropriate statistical methods for their analysis, the normality of these distributions will be assessed using the D'Agostino-Pearson omnibus test. Comparisons between groups for categorical variables will be performed using the chi-square test or Fisher's exact test, while continuous variables will be compared using the independent samples *t*-test or Mann–Whitney *U* test, depending on the results of the normality assessment.

To evaluate the performance of the AI- enhanced ECG algorithm in predicting PAF during SR, a comprehensive statistical analysis will be performed. The sensitivity, specificity, positive predictive value, negative predictive value, overall accuracy, and F1 score of the algorithm will be calculated by comparing its predictions with the reference to standard diagnoses. ROC analysis is conducted to determine the AUC, providing a comprehensive measure of the diagnostic performance of the algorithm. Optimal cutoff points are identified by maximizing the Youden index, which considers both sensitivity and specificity. To assess the performance of our AI model, we compared the AUCs from the current and previous datasets using the *z*-statistic (13). Additionally, measures including sensitivity, specificity, positive predictive value, negative predictive value, accuracy, and F1 scores from both datasets were analyzed using a two-sample proportion test.

Furthermore, subgroup analyses stratified by variables such as age, sex, and the presence of comorbidities will be conducted to evaluate the performance of the algorithm across diverse patient populations. Kaplan–Meier survival functions will be used to estimate the time to hospitalization, AF-related procedure outcomes, and mortality, with log-rank tests to compare patients with low and high risk of AF by AI. Using a multivariate Cox proportional hazards regression model, we will estimate the effect of AI-enhanced ECG multimorbidity on clinical outcomes after stratifying patients into groups according to AF probability as determined by AI, while adjusting for potential confounders such as age, sex, comorbidities, medications, and other clinically relevant factors. Additionally, we will conduct a subgroup analysis with 1:1 propensity score matching to adjust baseline characteristics for both groups to further understand their impact on our findings. The absolute standardized mean difference will be calculated to validate the propensity score matching. Moreover, adjustments will be made for various confounding factors, including age, sex, history of heart failure, hypertension, diabetes mellitus, previous ischemic stroke, transient ischemic attack, vascular events, and thromboembolism.

All statistical analyses will be performed using IBM SPSS Statistics 29 (IBM Corp., Armonk, NY, USA) and Python. Statistical significance is set at two-tailed *p* < 0.05. The necessary sample size for this study will be determined through power analysis using PASS 11 software (PASS 11, NCSS, LLC. Kaysville, Utah, USA) (13, 14).

## 3. Discussion

We will evaluate and validate the AI-enhanced ECG algorithm using a real-world large-scale dataset. Furthermore, we will examine the feasibility of incorporating the AI-ECG algorithm into clinical practice and evaluate its influence on AF-related patient outcomes.

This multicenter study involves 10 major university hospitals with a large sample size of over 50,000 cases. To date, no large-scale validation studies have been conducted at multiple institutions; hence, our study will provide a robust foundation for drawing meaningful conclusions with generalizable results.

Given the multifactorial nature and progressive characteristics of AF, it is important to consider various factors beyond the diagnosis itself, such as the timing of AF diagnosis, window period, and accurate comorbidity information. Therefore, accurate data labeling is essential for accurate and effective AI training (15, 16). One notable strength of our research design is the meticulous selection of data by specialized electrophysiologists from the cardiology departments of university hospitals. Within the landscape of AI-driven medical research, data labelling is a priority. Compared to previous studies, we do not solely rely on machine interpretations of the ECG readings. Instead, we will enhance the accuracy and reliability of our data through meticulous categorizing by electrophysiologists from all participating university hospitals.

Unlike traditional diagnostic methods that distinguish between AF and sinus rhythm only during ongoing “irregularly irregular” AF episodes, our AI model is uniquely designed to diagnose and predict AF occurrences even during SR. This breakthrough addresses a critical unmet medical need considering the sporadic and asymptomatic nature of PAF which often evades low detection yield by conventional methods, despite the risk of severe complications such as stroke and heart failure. Our methodology diverges fundamentally from existing approaches such as Holter monitors, smartwatches, and implantable cardiac monitors, not only in terms of data acquisition but also in the sophistication of our algorithm. Our AI model excels in identifying and amplifying specific ECG regions indicative of AF. Validated by cardiac arrhythmia experts rather than relying solely on machine-generated ECG readings, our approach has achieved a significantly higher F1 score, affirming its effectiveness and precision (9).

Our study will build on the significant findings of atrial remodeling and fibrosis in patients with recurrent AF after AFCA. Echocardiography and magnetic resonance late gadolinium enhancement can visualize these pathological changes (17). The promise of AI-enhanced ECG in detecting inflammation and fibrosis via the normal SR in patients with AF has been underscored. Recent AI-enhanced ECG data suggest that inflammation and fibrosis may be discerned through NSR on ECG in patients with AF (18). The use of AI-enhanced ECG-guided AF screening in SR has significantly improved AF detection rates (19). Our study aims to advance this field further. Our primary focus is not merely to improve AF diagnosis using AI, but to strengthen the connection with relevant clinical data. We anticipate that by implementing this comprehensive strategy, the capabilities of AI will be enhanced, with further improvements in the detection of AF and deeper understanding of the disease. We intend to further investigate the ability of AI-enhanced ECG to detect subtle changes in atrial inflammation and fibrotic remodeling. A central point of our investigation is the relationship between AI-enhanced ECG findings and the maintenance of SR in patients undergoing AF-related procedures. A recent study also highlighted the impressive performance of AI-enabled ECG in detecting AF on SR-ECG, showing increased efficacy when the algorithm incorporated SR-ECG after the index AF-ECG (20). This is consistent with the “window period” approach in our previous study.

Building on these findings, our study aims to report the ability of AI-enhanced ECG to accurately detect even subtle variations in atrial inflammation and fibrotic remodeling. Additionally, we will investigate the correlation between AI-enhanced ECG findings and the maintenance of SR in patients undergoing AF-related procedures. To gather relevant data, we will obtain ECG data at 3 and 6 month follow-ups, and every 6 months thereafter, to evaluate the outcomes of SR maintenance in patients undergoing AF catheter ablation, cryoablation, and DCCV.

We will also investigate the relationship between AI-enhanced ECG and antiarrhythmic medications, including their potential effects on ECG parameters. We aim to gain insights into the impact of antiarrhythmic agents on ECG patterns and identify any potential correlations between medication usage and AI-enhanced ECG findings (21–23). This research will contribute to a better understanding of the interaction between AI-enhanced ECG and antiarrhythmic therapies, ultimately informing clinical decision-making and optimizing treatment strategies for patients with AF. This analysis will enable us to assess the influence of medication use on the performance of AI-enhanced ECG in predicting AF. Examining the relationship between antiarrhythmic therapy and AI-enhanced ECG findings, this will provide valuable insights into the effect of medication status on the accuracy and effectiveness of AF prediction models.

The incidence of AF significantly increases in the population aged 65 years and above, and it is anticipated that this prevalence will increase further with the aging of the population (24). AF is asymptomatic in up to 40% of patients, but the risk of complications remains regardless of the presence or absence of symptoms (25). The yield of single time-point screening, including 12-lead ECG or handheld ECG, for unknown AF is 1%, rising to 1.4% for those aged 65 and older; importantly, only a minority of patients with a diagnosis of AF experience symptoms, and a significant proportion are undertreated (26). Our study aims to bridge a significant gap in the detection of AF. Conventional 12-lead ECG methods have shown limited detection yield, particularly in asymptomatic populations. However, in the EAST-AFNET 4 trial, early rhythm control was found to consistently improve clinical outcomes, reducing mortality rates, hospitalizations, and complications by 21%–24% in both asymptomatic and symptomatic patients newly diagnosed with AF (7, 8). These findings underpin the critical need for early detection and management of AF. This approach allows the promotion of early intervention to improve patient outcomes, aligning with the “early detection, early management” paradigm. The use of AI-enhanced 12-lead ECG shows potential for improving the early detection of AF, particularly in high-risk patients with paroxysmal episodes. In real-world clinical settings, patients identified as high-risk through AI-enhanced ECG could undergo intensive and prolonged Holter monitoring, along with rigorous outpatient follow-up, as a proactive approach to mitigate potential complications. This could represent a significant technological breakthrough in AF detection and screening that can be applied to the general population (5).

This study has some limitations. First, the retrospective design of our study may raise concerns about selection bias, documentation gaps, and data inconsistencies across hospitals. Our decision to use a retrospective approach was driven by the challenges of obtaining large-scale data that combines various clinical datasets, particularly in the field of AI-ECG research on AF. Notably, in the current NOAC era, discerning the relationship between AI-ECG and clinical outcomes such as stroke and systemic embolism associated with AF remains a significant hurdle, even within the design of a prospective study. Previous studies linking stroke to extensive clinical data used a retrospective dataset because of these challenges (27). Nevertheless, there remains a need for a large prospective clinical trial to validate the correlation with clinical outcomes. Second, our study will be conducted across a multicenter network of 10 major university hospitals. These variations can introduce heterogeneity into the data, potentially influencing the outcomes of our study. Although this enhances the generalizability of our findings to some extent, it is important to note that there may still be variations in patient populations, treatment protocols, and healthcare practices across different centers. Given the varying circumstances under which ECGs are performed in these settings, we used raw XML digital data to minimize the potential for bias and artifacts. Third, the algorithms used in AI are generally known as “black boxes” in terms of how they reach conclusions. This lack of transparency can be a significant barrier to the adoption of AI systems in medical settings, where understanding the reasoning behind recommendations is crucial for building trust and patient compliance. To address the challenges of AI interpretability in the arrhythmia field, we have incorporated Class Activation Maps (CAM) from methodologies like Grad-CAM, SHAP, and Dense Neural Networks, emphasizing explainable AI (28). Fourth, despite the impressive performance exhibited by deep learning algorithms, the challenge of effectively addressing false positives and false negatives to accurately identify appropriate therapies and forecast results remains a significant concern. Finally, in contemplating future applications of our AI-enhanced ECG algorithm, several suggestions warrant attention. Immediate plans include the development of an intuitive user interface designed to integrate seamlessly into routine clinical workflows, thereby augmenting the detection of AF. The sustainability of the effectiveness of the algorithm necessitates periodic re-training with updated data sets. Furthermore, addressing ethical considerations regarding data privacy and informed consent is imperative to ensure the responsible clinical integration of the algorithm. Addressing these critical factors is pivotal for the successful and ethical deployment of our technology in healthcare environments.

In conclusion, this large-scale, multi-center study aims to validate the real-world performance of our AI-enhanced ECG algorithm in diagnosing AF and to explore its association with AF-related clinical outcomes, thereby contributing to the improvement of AF detection and its potential integration into clinical practice.
